# Meat Quality Derived from High Inclusion of a Micro-Alga or Insect Meal as an Alternative Protein Source in Poultry Diets: A Pilot Study

**DOI:** 10.3390/foods7030034

**Published:** 2018-03-08

**Authors:** Brianne A. Altmann, Carmen Neumann, Susanne Velten, Frank Liebert, Daniel Mörlein

**Affiliations:** 1 Department of Animal Sciences, Division Animal Product Quality, University of Göttingen, 37075 Göttingen, Germany; brianne.altmann@agr.uni-goettingen.de; 2 Department of Animal Sciences, Division Animal Nutrition Physiology, University of Göttingen, 37077 Göttingen, Germany; carmen.neumann@agr.uni-goettingen.de (C.N.); susanne.velten@agr.uni-goettingen.de (S.V.); flieber@gwdg.de (F.L.)

**Keywords:** broiler, chicken, breast meat, sensory analysis, modified atmosphere packaging, Spirulina, black soldier fly, *Hermetia illucens*, *M. pectoralis superficialis*

## Abstract

The effects on meat quality resulting from alternative dietary protein sources (Spirulina and Hermetia meal) in poultry diets are studied to determine the overall suitability of these ingredients considering state-of-the-art packaging practices—highly oxygenated modified atmosphere packaging (HiOx MAP). We monitored standard slaughterhouse parameters, such as live weight, carcass weight, dressed yield, and pH at 20 min and 24 h post mortem. In addition, we studied the effects that 3 and 7-day storage in HiOx MAP has on the overall product physico-chemical and sensory properties. In addition to previously supported effects of HiOx MAP, we found that meat quality could be improved when Spirulina replaces 50% of the soy protein in broiler diets; however, this substitution results in a dark reddish-yellowish meat colour. On the other hand, the substitution with Hermetia larval meal results in a product that does not differ from the standard fed control group, with the exception that the breast filet has a more intense flavour that decreases over storage time. All-in-all Spirulina and Hermetia meal have the potential to replace soybean meal in broiler diets without deteriorating meat quality.

## 1. Introduction

Continued population growth and increasing income levels are driving up the demand for animal-based products and, in turn, is increasing the demand for animal feed resources [1]. Soybeans are a well-studied and widely applied as a source for protein in poultry and livestock diets. However, in recent years, concerns have mounted regarding the cultivation of soybean—particularly in topics such as world market power and the sustainability of production. Therefore, research institutions and industry alike are looking into the possible alternative animal feed protein sources available. The European Union (EU) relies on soybean imports to feed domestic poultry and livestock, with approximately 40% of animal feed protein originating from soy imports [2]. In addition, not only is the EU heavily dependent on soy imports, but China dominates the world import market by consuming approximately 41% of total world soy exports [3]. Furthermore, questions persist regarding the sustainability of soybean cultivation, primarily in the southern hemisphere. Prudêncio da Silva et al. [4] find that although deforestation is decreasing in Brazil, the previously incurred land use changes have led to secondary impacts, such as climate change and increased cumulative energy demand. Therefore, as a net importer of soy, the EU is conscious of their economic vulnerability and environmental responsibility.

This pilot study, focusing on poultry meat quality, is part of a larger project aimed at determining whether alternatives to soy can be incorporated into German meat production systems without having a negative effect on the overall product quality. Alternatives are deemed necessary in order to off-set soybean imports and decrease the European protein gap. Therefore, this study investigates the effects of two proteins that could be produced outside the arable farming system, focusing on one micro-algae source and one insect protein source, as exemplars. Spirulina (*Arthrospira platensis*) was chosen to partially replace soy because of its high crude protein content of 63% in dry matter (DM) [5] and the ability to cultivate it in photobioreactors or race-way ponds [2], which could reduce or at least limit the area required for cultivation in Europe. In addition, Spirulina contains antioxidants, such as β-carotene and vitamin E [6], which could have positive effects on meat physico-chemical parameters. *Hermetia illucens* L., also known as the black soldier fly, was also chosen as a prospective protein source for poultry diets because larvae also contain high amounts of crude protein [7] and could be fattened on numerous substrates, such as manure, cereals, and agro-food sector wastes [7,8], all of which are easily accessible in Europe, but are strictly regulated by the EU commission [9]. The conversion (recycling) of manure into a high quality animal feed [8] could be a notable advantage for Hermetia meal production; however, this is unlikely to be permitted within the EU. Unfortunately, to date in the EU, the use of animal originating feedstuffs is mostly prohibited in livestock production; nonetheless, insect feed is currently allowed in pet food and as of July 2017 has been approved for fish feed. Therefore, with continued legislative changes, Hermetia meal has the potential to be incorporated into poultry diets.

Although environmental and production sustainability are critical to ensuring the world’s food supply, it should not have to come at an expense to quality. Therefore, we endeavour to capture the multi-faceted concept of meat quality through physico-chemical and sensory testing in order to determine the effects of off-setting soy with Spirulina or Hermetia meal. Ultimately, meat quality is a combination of factors [10] that differ from region to region [11] and in context [12]. Physico-chemical characteristics are usually instrumentally evaluated using literature-agreed-upon methods and parameters [13]. Whereas, sensory testing is the only way to fully quantify the aroma and texture characteristics experienced, often in combination, by consumers. This more complex testing is necessary because, of course, the palatability of meat is a combination of factors that cannot (yet) be captured simultaneously by laboratory techniques. Finally, in order to evaluate whether the alternatively-fed products can be immediately integrated into current production chains in Europe, primarily Germany, meat quality is assessed with samples stored in the industry’s commonly practiced packaging [14]—highly oxygenated modified atmosphere packaging (HiOx MAP). The packaging type, length of storage time, and feed source can influence the flavour associated with aging or colour stability; therefore, in this pilot study, we investigate the impacts to physico-chemcial parameters and sensory properties when 50% of dietary soy protein is replaced by either *Arthrospira platensis* (Spirulina) or defatted dried *Hermetia illucens* larval meal (*Hermetia*) under current industrial packaging practices.

## 2. Materials and Methods

### 2.1. Animals and Diets

For this study, 132 Ross 308 male birds were raised on amino acid balanced diets where 50% of the soy-based protein was substituted by either Spirulina powder (*n* = 48) or Hermetia partially defatted larval meal (*n* = 48) in starter and grower diets; in the control group (*n* = 36) no soybean meal was substituted. The spirulina powder was sourced from Myanmar and had a moisture content of 3.4%. It was made up of 58.8% crude protein (DM) and 4.3% lipids (DM). The Hermetia meal was produced in Germany and the composition included 5.5% moisture, 60.5% crude protein (DM), and 14.1% lipids (DM). The main ingredients of the control diet during the starter, and respective (resp.) grower, period were wheat (32.9 resp. 37.6%), corn (16.4 resp. 18.8%), and soybean meal (39 resp. 32%). The diets were with amino acids supplemented according to the ideal amino acid ratio [15] and formulated to meet the energy and nutrient requirements of fast growing meat-type chickens according to current recommendations. Lysine level of the control diet was 1.25% and was 1.05% in the starter and grower periods. Table 1 outlines the diet composition for both starter and grower periods and the analysed diet nutritional content is listed in Table 2. The diets were created in reference to the Ross 308 nutritional specifications [16]. Further details to the procurement of Spirulina and Hermetia meal can be found in Neumann et al. [17].

The diets were fed ad libitum and animals had constant access to water. The animals were standardly kept according to article 4 of Germany’s Animal Welfare Regulation [19] on wood shaving covered floor pens (6 birds per pen; stocking density 5 birds per m^2^) at the Division Animal Nutrition Physiology, University of Göttingen, Germany. In total, 8 pens per Spirulina or Hermetia treatment group were housed, and 6 pens were fed the control diet. Animals were randomized amongst the pens prior to slaughter to reduce a possible housing effect later in the experimental design. At 35 days of age, the animals were slaughtered by certified personnel at the University of Göttingen poultry slaughterhouse, which is authorized according to article 4 of the European Union’s (EG) NR. 853/2004 [20]. Immediately following the slaughter, the *M. pectoralis superficialis* muscle (breast filet) was removed (excluding the *M. pectoralis profundus* muscle), and was cooled to 4 °C (ca. 5 h) until further processing, with the exception that pH and lean colour were monitored prior to cooling (see below).

### 2.2. Animal and Sample Management

**Figure 1:**
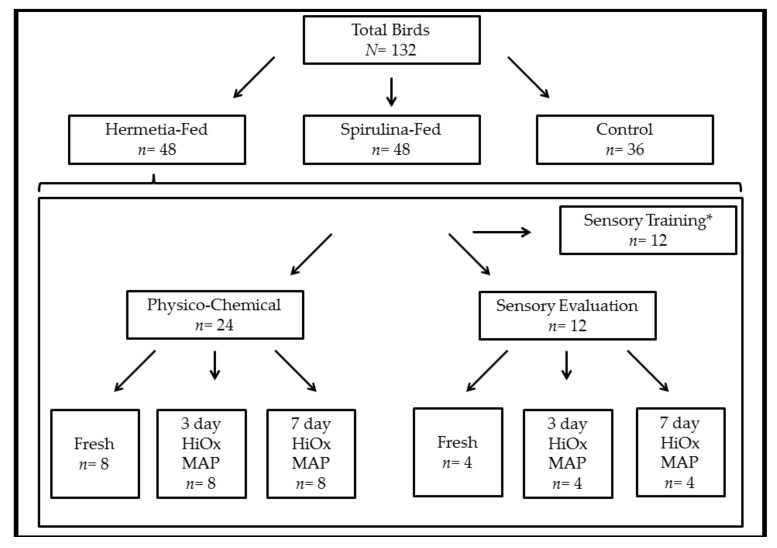
Allocation of birds (upper portion) and material within a treatment group (lower portion). * Only for Spirulina- and Hermetia-fed groups

In addition to the three feed treatment groups, the animals were further allocated into groups for physico-chemical or sensory testing, as can be seen in Figure 1. The first 12 carcasses per group (24 breast filets) were assigned for sensory testing and divided into 3 storage times so that 4 animals per feed treatment and storage time could be tested. The storage times were Fresh (not HiOx MAP packaged), 3 days, and 7 days. Two chicken filets derived from the same animal were packaged in polypropylene (PP) plastic trays (227/178/40 mm in dimension) lined with a moisture absorbent pad, heat-sealed with an oriented polyethylene terephthalate (OPET)/polypropylene (PP) film (<3 cm^3^/m^2^ 24 h bar oxygen transmission rate; <12 cm^3^/m^2^ 24 h bar carbon dioxide transmission rate; Dieter Seegers Haus der Verpackung GmbH, Osnabrück, Germany) and stored in an 80% O_2_/20% CO_2_ atmosphere. The packages were stored at 4 °C without illumination for the allotted time. Prior to sensory testing, the samples were vacuum-sealed in polyamide (PA)/polyethylene (PE) bags and frozen at −20 °C until further testing.

The next 24 carcasses per group were assigned for analytical meat quality testing and were also subsequently divided into 3 storage times: Fresh (*n* = 8), 3 days (*n* = 8), and 7 days (*n* = 8). The samples were packaged and stored as mentioned above. The following parameters were monitored: lean colour (20 min and 3 day or 7 day), pH (20 min and ultimate), lipid oxidation, storage loss (excluding Fresh samples), cooking loss, and shear force. The lipid oxidation samples were frozen at −70 °C prior to analysis and the right breast filet was frozen at −20 °C prior to cooking loss and subsequent shear force analysis. The additional animals in the Spirulina-fed (*n* = 12) and Hermetia-fed (*n* = 12) treatment groups (indicated with * in Figure 1) were also probed for pH_20min_ and pH_24h_ in the left filet, and the right filets were allocated for sensory training.

### 2.3. Physico-Chemical Characteristics

The pH was measured at 20 min post mortem and an ultimate pH measurement (pH_u_) was taken at 24 h for the Fresh samples, and at 3 days or 7 days for the respective HiOx MAP samples. The measurements were taken with a portable pH meter equipped with a glass electrode and metal thermometer electrode (Knick Portamess 911, Berlin, Germany). The pH meter was calibrated every session prior to use using commercial pH 4 and 7 buffer solution standards (Merck, Darmstadt, Germany) at room temperature. The pH was measured by inserting the electrode completely into the superior portion of the left breast filet muscle. An accompanying thermometer was inserted alongside the electrode approximately 1 cm away. The lean colour was measured at 20 min post mortem and at 3 days or 7 days immediately after opening the package; no blooming time was allowed so that colour was recorded as close to consumers’ perception through the packaging. CIELAB coordinate measurements were recorded and the values used in analysis were derived across the average of three measurements taken on the ventral side of the right breast filet using a portable spectrophotometer (model: CM 600d, Konica Minolta, Tokyo, Japan) with diffused illumination, an 8° viewing angle, and a silicon photodiode array as the detector. The spectrophotometer was calibrated using a white and black prop provided by the manufacturer prior to every session. In order to determine if the protein feed had an effect on meat composition, or whether meat composition could be significantly correlated with shear force values, breast filets were cleaned of excess subcutaneous fat, homogenized, and meat composition parameters were analysed using a Foss FoodScan^TM^ according to Anderson [21]. To determine storage loss, the breast filets allocated for HiOx MAP were weighed prior to packaging and immediately after being removed from the package. Storage loss was expressed as the percent of weight loss over time compared to the initial sample weight. To determine cooking loss, the right breast filet, trimmed of exterior fat, was cooked sous vide for 60 min. The samples were vacuum-sealed in PA/PE bags and placed in a pre-heated hot water bath instrument (incubation/deactivation bath, Gesellschaft für Labortechnik mbH (GFL), Burgwedel, Germany) set to 77 °C. Samples were kept submerged and separated during cooking. Within the 60 min, the samples achieved 75 °C core temperatures, as determined with a preliminary study. After cooling to room temperature, the samples were weighed and cooking loss was expressed as the percentage of the initial weight. After determining cooking loss, the samples were stored at 4 °C for 24 h prior to shear force measurements. Shear force was determined according to Xiong et al. [22] with the following modifications: a TA.XTplus Texture Analyser (Stable Micro Systems, Surrey, UK) was set to a penetration depth of 15 mm to accommodate for thinner samples and a 50 N load cell was used. Each sample was tested 3 times and statistical analysis was conducted with the mean value across the 3 measurements. Shear force was measured as the highest peak (N).

### 2.4. Lipid Oxidation

In order to determine the extent of lipid oxidation over time, the 2-thiobarbituric acid reactive substances (TBARS) method was conducted according to Bruna et al. [23]. Results were recorded in terms of μg of malonaldehyde (MDA)/g of sample.

### 2.5. Sensory Analysis

Conventional profiling was used to determine the appropriate attributes to be later evaluated. Appropriate attributes were determined prior to evaluation by the assessors. In eight two-hour training sessions, the panel legitimated and defined the attributes unique amongst the products. These 19 attributes were used to evaluate the products in appearance, odour, taste and flavour, texture, and aftertaste. Overall odour intensity, animal or barn odour, metallic odour, cooked chicken odour, colour intensity (light-dark), visual elasticity assessment, fibrous appearance, overall flavour, sweet taste, sour taste, bitterness, metallic flavour, chicken flavour, overall aftertaste, hardness, juiciness, tenderness, adhesiveness, and crumbliness were evaluated (see Appendix A for more information).

The samples were cooked sous vide exactly as described for cooking loss above. The core temperature of every breast filet was checked to ensure that every sample was ca. 75 °C using a Testo 926 digital probe thermometer (Testo SE & Co. KGaA, Lenzkirch, Germany). The breast filets were then cut into 1 cm by 1cm pieces. Samples were served immediately on warmed plates and assigned with a 3 digit randomly allocated code. The evaluations took place in the University of Göttingen sensory laboratory, which is in compliance with international standard-ISO 8589 [24].

The trained panel consisted of ten assessors, who voluntarily provided written informed consent and were selected and trained according to ISO 8586-1 [25]. The panel evaluated chicken breast filet products differing in protein feed type (Spirulina, Hermetia, Control) and storage time (Fresh, 3 day HiOx MAP, 7 day HiOx MAP). In total, the nine products, derived from the three-by-three design, were evaluated in duplicate by each assessor over three one-hour sessions, where each assessor evaluated one sample for six of the nine products per session. The assessors evaluated the samples in a sequential monadic manner following four set orders that were randomly allocated, and a maximum of three assessors received the same set order during one session. The measurements were recorded electronically on 9 cm unmarked scales, a point system from 0 to 100 was stored behind the scale for later statistical analysis, using EyeQuestion survey software (Logic8 BV, Elst, The Netherlands). No assessor evaluated two samples from the same bird.

### 2.6. Statistical Analyses

The statistical analyses were carried out using SPSS (Version 24.0, IBM Corporation, Armonk, NY, USA) statistical software. One-way ANOVAs were conducted to determine the effect of feed on the carcass and meat quality parameters, such as live weight, carcass weight, the breast filet yield per animal, meat composition and pH_20min_ and pH_24h_. Factorial ANOVA was carried out to determine the effects that feed, storage time (main effects), and a possible interaction effect (Feed × Storage) had on lean colour, lipid oxidation, storage loss, cooking loss, and shear force. The sensory data were evaluated using a linear mixed model with the sensory attributes as the dependent variables, feed, storage time in HiOx MAP, and a Feed × Storage interaction term as the fixed effects, and the random effects were listed as assessor and animal. Post Hoc tests were conducted using the Fischer’s least significant difference (LSD) technique. Significant differences were determined where *p* < 0.05. In addition, effect size was calculated to account for possible Type 2 statistical errors, which could be likely due to the experiment’s small sample size; by reporting the effect size one would nonetheless be able to compare our results to other studies and to interpret the relevance of effects as opposed to significance with the latter being substantially affected by sample size (increasing *n* ultimately leads to significance, even though the effect size is equally small as with a low *n*). Either Cohen’s *d* for carcass performance parameters, where either Spirulina or Hermetia treatment group results were compared with the control, or partial eta-squared were calculated to estimate the effect size for physico-chemical data. Effect size was interpreted according to Cohen [26], where a small size is deemed to be 0.2 > 0.5, a moderate effect size is 0.8 < 0.5, and a large effect size is above 0.8. These values also correspond with the interpretation applied by Batorek et al. [27] in order to ascertain that the parametrical differences are derived from the treatments and not from zootechnical parameters, such as breast filet weight. Further, to maintain certainty that the significant differences were derived from the treatments, Pearson’s r was also calculated between the weight of one breast filet and the corresponding dependent parameters—lean colour (L*, a*, b*), lipid oxidation (TBARS), storage loss, cooking loss, and shear force, and between shear force values for Fresh samples and intramuscular fat (IMF) or moisture as dependent factors. A correlation was considered significant at a level of 0.05 using a two-tailed test.

## 3. Results

### 3.1. Physico-Chemical Results

There are only minor differences in carcass performance indicators between the feed treatment groups. Hermetia-fed birds were larger in size, which is accounted for by the statistically significant differences described in Table 3 for live weight and carcass weight. However, this difference in weight did not result in an overall increase in breast filet yield. Furthermore, the pH_20min_ and pH_24h_ values are significantly different between the different feed groups, with the control group having the highest pH values directly after slaughter, but the Spirulina-fed samples maintain a higher pH after 24 h.

Regarding the breast filet quality, the estimated marginal means for the analysed quality characteristics are listed in Table 4. In terms of feed, nearly all differences are accounted for by the Spirulina feed. These samples were darker (L* value), redder (a* values), and more yellow (b* values) in colour compared to the Hermetia-fed and control groups. The Spirulina-fed samples lost 0.5% less moisture while being stored and were able to retain that water even after cooking, as is shown by the nearly 2% decrease in cooking loss values compared to the Hermetia-fed group and 4% decrease in values compared to the control group. The Hermetia-fed values remained similar to those of the control group across all the studied parameters. TBARS and shear force values were not significantly different across the feed groups. In addition, breast filet weight (g) correlated minimally only with the lean colour parameters L* (*r* = 0.589 with *p* < 0.01) and b* (*r* = 0.319 with *p* < 0.05).

Concerning the effect of HiOx MAP storage, the results are not always so clear-cut. To start, the storage in HiOx MAP has an effect on the lightness values (L*) with a steady increase in the values (lightness) over the three groups. HiOx MAP also appears to increase the a* and b* values; however, these values fall slightly between day 3 and day 7. The TBARS values are not significantly different between the Fresh samples and the 3 day HiOx MAP samples; however, the values do significantly increase for 7 day HiOx MAP samples. The HiOx MAP samples were significantly different from the Fresh samples in terms of cooking loss. The HiOx MAP samples lost 3% less moisture during cooking than their Fresh counterparts. Finally, the storage losses where similar between the HiOx MAP groups, despite the four day difference in storage times, and the shear force values were similar across the groups and not significantly correlated to meat composition parameters—IMF and moisture content.

**Figure 2:**
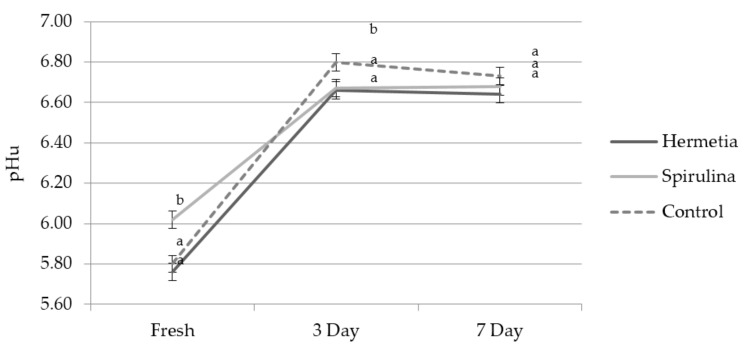
Interaction effect of feed and storage times (*p* = 0.002) on pH_u_ (mean, SE); superscript letter a–b denote statistical differences between the feed groups.

Interestingly, the only significant interaction effect (Feed × Storage) is for pH_u_ (*p* = 0.002). Here we can see that after the initial fall of the pH_20min_ values to the pH_24h_ values (see Table 1), the values climb back up again over time in HiOx MAP (illustrated in Figure 2). The values are the highest for the control group compared to the two treatment groups, yet remain relatively stable between the 3 and 7 day tests for all HiOx MAP samples.

### 3.2. Sensory Results

Most attributes were not discernibly differentiated by the trained panel. However, the assessors determined that the feed significantly affected the hardness (*p* < 0.003) and tenderness (*p* < 0.008); storage time significantly affected elasticity when samples were stored up to 7 days (*p* < 0.024), and the interaction effect Feed × Time (*p* < 0.002) was significant for the overall flavour intensity. The effect of feed on metallic flavour was nearly significant (*p*-value = 0.051). Please refer to Table 5 to see the quantitative differences discerned. Generally, the Spirulina-fed samples were less metallic in flavour and the two alternative feed groups were softer and more tender than the control group.

**Figure 3:**
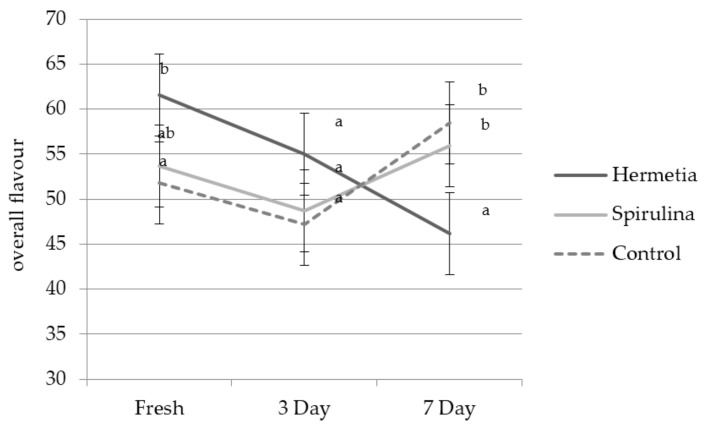
Interaction effect between feed and storage time on overall flavour (*p* = 0.002); superscript letter a–b denote statistical differences between the feed groups.

The interaction between feed type and HiOx MAP storage times is significant, and as illustrated in Figure 3 (*p* = 0.002), storage appears to have different effects depending on the feed type. For example: HiOx MAP storage appears to decrease the flavour intensity over time for Hermetia-fed samples, but not for Spirulina-fed or control samples. However, for all three groups, the 3 day HiOx MAP samples are less intense than the Fresh samples, and for the Spirulina-fed and control samples, the intensity climbs again after four additional days of aging in HiOX MAP. Finally, 7 day HiOx MAP (estimated mean of 34.0) results in a product that is less elastic compared to the Fresh (estimated mean of 41.9) and 3 day HiOx MAP (estimated mean of 41.3) breast filets (*p* = 0.024; SE= 5.41).

## 4. Discussion

The European protein gap is one reason why alternative protein sources should be widely studied [28]. Therefore, understanding the effects on meat quality from soybean meal substitution in poultry diets is important when considering what this elemental change could mean for packers, retailers, and consumers down the supply chain. At a quick glance, our results for the 50% substitution of soy-based protein with Spirulina or Hermetia larval meal, show very modest or no changes in the meat quality for many of the relevant parameters. The lack of correlation between breast filet size and the physico-chemical parameters shows that the significant differences listed above are likely not due to zootechnical differences in size between the groups, but rather due to the feed type and storage length treatments. The exceptions are L* and b*, where the larger filet size could influence the measureable differences; although if the filet size was the main influencing factor, then one would expect that the Hermetia-fed would be the diverging group, not the Spirulina-fed. The effect sizes, according to Cohen’s *d*, imply that diet has a moderate effect on the carcass performance parameters, such as carcass weight and breast yield; however, the diet has a much larger effect on the pH value, especially for the Spirulina treatment group after 24 h, and type 2 statistical errors are likely not a factor with which to be concerned. According to the partial eta-squared parameters, it could be said that although feed may have a modest effect on meat quality, the type of packaging in conjunction with length of storage, in the end, plays a larger role in influencing meat quality. Although most of the results may be negligible, some of the deviations from the standard soy-fed control group should be well considered (lean colour) and appreciated (carcass weight) prior to fully incorporating alternative protein sources into poultry diets.

### 4.1. Spirulina in Poultry Diets

The incorporation of Spirulina results in a breast filet with a higher pH value at 24 h post mortem. In addition, storage and cooking losses decreased compared to the other two treatment groups, and this is likely related to the higher pH value. Although in our study the diets were only supplemented to balance dietary amino acids, increased dietary Lysine levels in diets has also resulted in similar findings of increased pH values and decreased storage losses [29]. Nonetheless, the result is improved meat quality, with a large effect size as according to the Cohen’s *d* value. Reduced cooking loss should result in a breast filet that is expected to be ‘juicier’ [30] and more tender in texture [31]; however, sometimes these differences are first perceived by trained assessors when large differences of over 10% occur [32]. The trained panel in our study did not evaluate the Spirulina-fed breast filets as being juicier; however, Spirulina-fed breast filets are rated as being the most tender and the softest. Albeit, these traits are not significantly different as compared to those of the Hermetia-fed breast filets. Spirulina-fed breast filets also had the lowest values for metallic taste. Metallic flavour is usually considered an off-flavour in poultry products, such as white meat breast filets, given that it is a typical descriptor for game [33] and beef [34] products. Indeed, Brunton et al. [35] list high levels 1-octen-3-one as having a metallic odour associated with off-flavours in turkey breast meat, and Jayasena et al. [36] list metallic flavour as an off-flavour resulting from lipid oxidation in chicken meat. Therefore, reduced scores can be interpreted as having a positive impact on the flavour of Spirulina-fed breast filets.

The largest and most readily noticeable difference of Spirulina-fed breast filets is the significant intensive colour. Spirulina-fed breast filets are darker, redder, and more yellow in colour than the other two treatment groups tested in this study. These results are in alignment with Venkataraman et al. [37] and Toyomizu et al. [38], who noted the distinct colour of the breast filet when Spirulina was incorporated into poultry diets. The distinctive colour is not just apparent in instrumental spectrophotometer measurements, but also to the naked eye in the raw state when looking at the entire muscle. Surprisingly, when the breast filets are cooked, a colour difference between the products was not discernible by our trained panel, although we must note that it may be difficult to detect colour differences with only a 1 cm^2^ surface area. The more intense colour is likely a result of the high amounts of carotenoids in Spirulina [6]. Holman and Malau-Aduli [39] believe this darker colour could be advantageous when feeding Spirulina to livestock, because meat colour is one of the most important quality indicators perceived by consumers [40,41,42]. However, precisely for this reason, consumer acceptance of and consumers’ response to intensely pigmented poultry products needs to be studied, prior to incorporating Spirulina-fed products into the current production chain.

### 4.2. Hermetia in Poultry Diets

Hermetia larval meal could be used to substitute soybean meal, with minimal effects reaching the packers, retailers, or consumers. In fact, as with Spirulina, Hermetia feed could improve the overall meat quality. Hermetia animals were about 150 to 200 g heavier than their peers, and this translated into heavier dressed carcasses as well. Although the diets were calculated to be constant in caloric intake, the Hermetia meal diet was substantially higher in crude protein and ether extract (lipids), especially compared to the other treatment group, Spirulina. Therefore, it is likely that this effect is more due to the imbalances between the diets, than an inherent effect caused by Hermetia meal itself. The only other parameter that differed from the control group concerns the overall flavour intensity of Hermetia-fed breast filets. Here, a trend is noticed, where the Fresh Hermetia-fed breast filets score as the most intensive flavour; however, the intensity decreases when in HiOx MAP and over time. Although not fed with Hermetia, but rather house fly larvae, Gawaad and Brune [43] found that broiler chickens raised on (non-defatted) house fly and blow fly larvae meal had a unique smell and an intensive taste. However, this stronger taste does not need to be taken as a reduction in meat quality, because as Sheppard et al. [7] point out, some consumers prefer stronger tasting meat.

### 4.3. Impact of Storage Time

As previously mentioned, we packaged samples in HiOx MAP for up to 7 days, because in Germany, the country of study, this is commonly practiced by the industry for breast filet packaging and retailing [14]. We wanted to ensure that the alternatively-fed breast filets could be submitted to these conditions without reduced quality compared to the control product. In that regard, it appears that neither the Spirulina-fed nor the Hermetia-fed breast filets are negatively affected by HiOx MAP more so than the control. That being said, HiOx MAP storage over time did have some effects on overall product quality. For example, HiOx MAP increased the lightness, redness, and yellowness of the samples; although the samples were not able to maintain the complete colour change from day 3 until day 7. The values sank slightly over time. Given that the main reason for using HiOx MAP is to stimulate a more intensive red colour [42], this is not an unexpected result. Other effects include: increased TBARS values between the 3 day and 7 day HiOx MAP samples. This is expected granted that a HiOx environment induces lipid oxidation [44,45,46,47,48]. In addition, HiOx MAP breast filets had lower cooking losses compared to breast filets that were not packaged, but directly frozen to be analysed. This could be in part due to the additional storage time of 3 and 7 days, not specifically due to HiOx MAP. The additional aging time allowed the samples to lose about 3% of their weight; the difference in cooking loss between the HiOx MAP and Fresh breast filets is about 3%. The HiOx MAP breast filets did not lose more moisture if they were packaged for 7 compared to only 3 days. These results are similar to those of Delles and Xiong [44], who found limited changes in water-holding capacity between 4 and 14 days for HiOx MAP pork. It appears that there is a limit to storage loss amounts in HiOx MAP over time, *ceteris paribus*. Finally, 7 days of HiOx MAP impacted the elasticity of the cooked breast filets. The longer packaged breast filets were not as ‘springy’ when compressed with a fork and released to retake its original form. This could be due to protein oxidation [47] and a probable increase in myofibrillar deterioration [44].

### 4.4. Interaction Effects Feed × Storage

There are two significant interaction terms: (1) for ultimate pH and (2) for overall flavour intensity. First, the general increase in pH value in a HiOx MAP over time is supported by [43] who found that HiOx MAP increases pH values over time in pork products. However, the Spirulina- and Hermetia-fed breast filets appear to have more stable pH values in HiOx MAP compared to the control group. Although microbial indicators were not monitored in this study, according to Allen et al. [49], this could indicate that Hermetia- and Spirulina-fed breast filets could have a longer shelf-life. Secondly, the interaction term between the Hermetia-fed breast filets in HiOx MAP differs from that of the control breast filets. The Hermetia-fed breast filets taste less intense when packaged in HiOx MAP and the intensity decreases over the time that they are packaged. The Spirulina-fed and the control breast filets also decrease in flavour intensity when packaged, but the flavour intensity increases again from day 3 to day 7. This increase in intensity over time is expected granted that lipid oxidation increased between 3 and 7 days and lipid oxidation often leads to off-flavours in chicken meat [36].

### 4.5. Looking Forward

There are many parameters that should continue to be monitored in order to fully understand the impact that these alternative feeds have on the breast filet end product. The fatty acid composition and nutritive content, including vitamins, should be studied to determine if there are any nutritive benefits to the consumer. Studies should also monitor the pH stability of alternatively-fed products in turn with microbiological data to resolve whether these products could have longer shelf-lives than the standard fed product. In addition, focus should not be taken from determining the consumer response to products that potentially taste stronger or have a more intense colour at the point of purchase. Therefore, further research should include purchasing experiments based on poultry product colour, and more sensory testing with Hermetia-fed products should be executed. Despite the research gaps still present, we remain convinced that Hermetia and Spirulina are two good candidates to replace soybean meal in poultry diets.

## 5. Conclusions

Spirulina-fed breast filets generally have a higher pH, a higher water-holding capacity during storage and cooking, and have a reduced (metallic) off-flavour. The majority of the differences may be inconsequential; however, the intense colour of the Spirulina-fed breast filets should be further researched prior to incorporating Spirulina into poultry diets at a large scale. Hermetia-fed animals and carcasses are heavier and do not differ from the control group in other physico-chemical parameters. The breast filet has a more intense flavour when not HiOx packaged or aged over time, yet it remains to be seen if these differences are discernible by consumers. Based on their nominal effects on meat quality, Spirulina and Hermetia larval meal remain two potential protein alternatives for poultry diets.

## Figures and Tables

**Table 1:** Ingredient composition of experimental diets (g/kg as fed).

Ingredients/Diets	Starter Period (1–21 day(s))			Grower Period (22–34 days)		
	Hermetia	Spirulina	Control	Hermetia	Spirulina	Control
Wheat	358.3	377.9	328.8	402.6	416.8	375.8
Corn	179.2	189.0	164.4	201.3	208.4	187.9
Soybean meal	195.0	195.0	390.0	160.0	160.0	320.0
Insect meal	145.4	-	-	119.0	-	-
Algae meal		118.2			97.0	
Soybean oil	78.5	78.5	78.5	78.5	78.5	78.5
Premix *	10.0	10.0	10.0	10.0	10.0	10.0
CaHPO_4_	12.0	12.0	11.0	8.0	10.0	10.0
CaCO_3_	9.9	9.1	11.0	8.0	8.0	9.0
NaCl	1.7	1.7	3.0	2.0	2.0	3.0
Wheat starch	-	-	-	3.0	3.0	-
TiO_2_	-	-	-			3.0
l-Lysine∙HCl	3.2	4.4	1.3	2.4	3.5	0.8
dl-Methionine	4.1	3.5	2.0	3.0	2.5	2.0
l-Threonine	0.6	-	-	0.4	-	-
l-Arginine	2.2	0.7	-	1.4	0.1	-
l-Valine	-	-	-	0.5	0.2	-

* Added per kg of final diet: 2.1 g calcium, 0.8 g sodium, 5000 IU vitamin A, 1000 IU vitamin D3, 30 mg vitamin E, 2.6 mg vitamin B1, 4.8 mg vitamin B2, 3.2 mg vitamin B6, 20 μg vitamin B12, 3 mg vitamin K3, 50 mg nicotinic acid, 10 mg calcium pantothenate, 0.9 mg folic acid, 100 μg biotin, 1000 mg choline chloride, 50 mg Fe as iron-II-sulfate, monohydrate, 15 mg Cu as copper-II-sulfate, pentahydrate, 120 mg Mn as manganese-II-oxide, 70 mg Zn as zinc oxide, 1.4 mg I as calcium iodate, hexahydrate, 0.28 mg Se as sodium selenite, 0.55 mg Co as alkaline cobalt-II-carbonate, monohydrate, and 100 mg butylhydroxytoluol.

**Table 2:** Analysed nutrient content of experimental diets (crude nutrients g/kg dry matter (DM)).

Diets	Starter Period (1–21 day(s))			Grower Period (22–34 days)		
	Hermetia	Spirulina	Control	Hermetia	Spirulina	Control
Crude protein	259.3	241.4	249.5	230.9	207.2	220.2
Ether extract	131.1	116.6	111.6	131.4	118.4	112.8
Crude fibre	47.1	31.1	45.2	41.7	30.4	40.4
Crude ash	60.4	59.2	65.6	56.5	53.5	61.6
N-free extract	502.1	551.7	528.1	539.5	590.5	565
AME_N_ (MJ/kg DM) *	15.3	15.4	14.4	15.6	15.6	14.8

* N corrected apparent metabolizable energy, calculated according to WPSA [18].

**Table 3:** Means and standard deviation (SD) with Cohen’s *d* for carcass performance parameters: live weight, carcass weight, breast filet yield, protein, intramuscular fat (IMF), moisture, and pH values at 20 min and 24 h post mortem.

Parameters	Hermetia	SD	Cohen’s *d*	Spirulina	SD	Cohen’s *d*	Control	SD
Live weight (g)	2329 ^a^ (*n* = 48)	322	0.509	2121 ^b^ (*n* = 48)	218	0.181	2169 ^b^ (*n* = 36)	306
Carcass weight (g)	1789 ^a^ (*n* = 48)	261	0.476	1577 ^b^ (*n* = 48)	175	0.465	1672 ^b^ (*n* = 36)	230
Breast filet yield (%)	20.4 ^a^ (*n* = 36)	1.9	0.205	20.0 ^a^ (*n* = 36)	2.0	0.400	20.8 ^a^ (*n* = 24)	2.0
Protein (% breast filet)	21.07 ^a^ (*n* = 8)	0.27	0.99	21.60 ^a^ (*n* = 8)	0.64	1.56	20.52 ^a^ (*n* = 8)	0.74
IMF (% breast filet)	3.41 ^a^ (*n* = 8)	0.45	0.02	3.12 ^a^ (*n* = 8)	0.50	0.47	3.40 ^a^ (*n* = 8)	0.68
Moisture (% breast filet)	73.88 ^a^ (*n* = 8)	0.43	0.62	73.62 ^a^ (*n* = 8)	0.71	0.87	74.29 ^a^ (*n* = 8)	0.83
pH_20min_	6.65 ^b^ (*n* = 36)	0.12	0.917	6.67 ^b^ (*n* = 36)	0.12	0.750	6.76 ^a^ (*n* = 24)	0.12
pH_24h_	5.80 ^a^ (*n* = 20)	0.18	0.00	6.06 ^b^ (*n* = 19)	0.13	1.852	5.80 ^a^ (*n* = 8)	0.15

Sample size indicated in brackets; superscript letter a–b indicate statistical differences between feed groups (*p* < 0.05); Cohen’s *d* comparisons are between the respective treatment group and control group.

**Table 4:** Estimated marginal means with standard error (SE) and effect size denoted by partial η^2^ for colour, lipid oxidation (2-thiobarbituric acid reactive substances (TBARS)), storage loss, cooking loss, and shear force.

Parameters	Feed Groups			Partial η^2^	Storage Time			Partial η^2^	SE
	Hermetia	Spirulina	Control		Fresh	3 Day	7 Day		
L*	55.60 ^a^	53.01 ^b^	55.40 ^a^	0.243	51.10 ^f^	54.92 ^e^	58.00 ^d^	0.648	0.454
a*	2.00 ^b^	3.48 ^a^	2.43 ^b^	0.212	0.85 ^f^	4.04 ^d^	3.03 ^e^	0.554	0.261
b*	11.15 ^b^	12.31 ^a^	11.33 ^b^	0.131	8.65 ^e^	13.15 ^d^	12.99 ^d^	0.716	0.286
TBARS (μg/g)	0.106 ^a^	0.095 ^a^	0.098 ^a^	0.006	0.081 ^e^	0.084 ^e^	0.134 ^d^	0.150	0.013
Storage loss (%)	3.00 ^a^	2.48 ^b^	3.04 ^a^	0.147	-	2.72 ^d^	2.96 ^d^	0.035	0.134
Cooking loss (%)	31.80 ^a^	29.00 ^b^	33.05 ^a^	0.169	33.29 ^d^	30.29 ^e^	30.25 ^e^	0.125	0.821
Shear force (N)	10.86 ^a^	11.16 ^a^	11.80 ^a^	0.060	11.01 ^d^	11.58 ^d^	11.21 ^d^	0.058	0.336

*n* = 24, except for storage loss (*n* = 16); superscript letter a–b indicate statistical differences between the feed groups (*p* < 0.05); superscript letter d–f indicate statistical differences between storage times (*p* < 0.05).

**Table 5:** Estimated means with standard error (SE) and effect size (partial η^2^) for the statistically significant sensory attributes (metallic flavour, hardness, and tenderness) according to feed type.

Sensory Attribute	Hermetia	Spirulina	Control	Partial η^2^	SE
Metallic flavour (not metallic to strongly metallic)	20.4 ^a^	15.8 ^b^	17.1 ^a^	0.223	3.90
Hardness (soft to hard)	28.8 ^b^	25.0 ^b^	40.8 ^a^	0.300	4.93
Tenderness (tender to tough)	30.8 ^b^	24.4 ^b^	47.1 ^a^	0.371	6.05

The table values are based on the 9 cm scale which was quantified from 0 to 100; superscript letter a–b denote statistical differences between the feed groups.

## Appendix A

**Table A1:** Sensory attributes identified and evaluated by trained assessors for characterizing chicken breast filets; scale from 0 to 100.

Attribute	Description	Sense	References *
1. Colour intensity	The intensity of the colour from white to dark beige	Visual	N/A
2. Elasticity	How far the sample can be pressed and still return to original form	Visual	<30: spreadable cheese
			50: white bread
			100: wine gum
3. Fibrousness	Fibre thickness on the product surface	Visual	<30: human hair
			<70: ca. 2 mm thick
4. Overall odour	The intensity of the smell when product held 2 cm from nose	Smell	N/A
5. Animal/Barn odour	Intensity of faecal, barn, or animal odour	Smell	Skatol
6. Metallic odour	Intensity of metal odour	Smell	old oxidized coins
7. Cooked chicken odour	Intensity of the odour from chicken meat in soup	Smell	cooked chicken juice
8. Overall flavour	Intensity of the overall flavour	Taste	N/A
9. Sweet taste	Intensity of sweetness	Taste	sucrose solutions
			50: 6 g/L
			100: 18 g/L
10. Sour taste	Intensity of sour taste	Taste	citric acid solutions
			50: 0.28 g/L
			100: 0.4 g/L
11. Bitter taste	Intensity of bitterness	Taste	caffeine solution
			50: 0.21 g/L
			100: 0.3 g/L
12. Metallic flavour	Intensity of metallic taste, such as serum or old coins	Taste	N/A
13. Chicken flavour	Intensity of taste similar to chicken soup	Taste	chicken soup broth
14. Aftertaste	The intensity of the aftertaste after swallowing	Taste	N/A
15. Hardness	The force needed to bite through the sample	Texture	<30: spreadable cheese
			50: gouda cheese
			100: Werther’s Original
16. Juiciness	Amount of fluid released from product upon first bite	Texture	<30: raw carrot
			50: cucumber
			100: orange
17. Tenderness	The degree that the sample remains in one piece while chewing	Texture	<30: raw carrot
			50: gouda cheese
			100: hard candy
18. Adhesiveness	The degree the product sticks to your teeth; force needed to pull teeth apart	Texture	50: spreadable cheese
			100: peanut butter
19. Crumbliness	Number of particles in mouth directly prior to swallowing	Texture	N/A

* Quantitative/qualitative.
